# Cardiac Amyloidosis with Discordant QRS Voltage between Frontal and Precordial Leads

**DOI:** 10.3390/medicina57070660

**Published:** 2021-06-27

**Authors:** Csilla-Andrea Eötvös, Roxana-Daiana Lazar, Iulia-Georgiana Zehan, Erna-Brigitta Lévay-Hail, Giorgia Pastiu, Mihaela Pop, Anca Simona Bojan, Sorin Pop, Dan Blendea

**Affiliations:** 1 “Niculae Stancioiu” Heart Institute, 400001 Cluj-Napoca, Romania; csilla.andrea18@gmail.com; 2 Department of Medicine, Faculty of Medicine, University of Medicine and Pharmacy “Iuliu Hatieganu”, 400012 Cluj-Napoca, Romania; daiana.pocol@yahoo.com (R.-D.L.); iuliazehan@gmail.com (I.-G.Z.); hail.brigitta@gmail.com (E.-B.L.-H.); giorgia23pastiu@yahoo.com (G.P.); ancasbojan@yahoo.ca (A.S.B.); popsorin98@gmail.com (S.P.); 3 Cluj County Emergency Hospital, 400000 Cluj-Napoca, Romania; 4 Schulich Heart Research Program, Sunnybrook Research Institute, Toronto, ON M4N 3M5, Canada; mihaela.pop@utoronto.ca; 5 Ion Chiricuta Oncology Institute, 400015 Cluj-Napoca, Romania

**Keywords:** amyloidosis, cardiomyopathy, low voltage

## Abstract

Among the different types, immunoglobulin light chain (AL) cardiac amyloidosis is associated with the highest morbidity and mortality. The outcome, however, is significantly better when an early diagnosis is made and treatment initiated promptly. We present a case of cardiac amyloidosis with left ventricular hypertrophy criteria on the electrocardiogram. After 9 months of follow-up, the patient developed low voltage in the limb leads, while still maintaining the Cornell criteria for left ventricular hypertrophy as well. The relative apical sparing by the disease process, as well as decreased cancellation of the opposing left ventricular walls could be responsible for this phenomenon. The discordance between the voltage in the frontal leads and precordial leads, when present in conjunction with other findings, may be helpful in raising the clinical suspicion of cardiac amyloidosis.

## 1. Introduction

Amyloidosis encompasses a heterogenous group of protein-folding anomalies, that lead to organ dysfunction due to accumulation of amyloid fibrils, originated from several precursor proteins, in the extracellular space. Immunoglobulin light chain (AL) amyloidosis is characterized by a monoclonal (κ or λ) proliferation of plasma cells, in which heart involvement represents the most severe form of the disease [1,2]. In addition to infiltration of myocardial tissue leading to structural and functional abnormalities, tissue deposits also interfere with renal and neural function, whilst gastrointestinal and hepatic infiltration appear less frequently. Cardiac amyloidosis (CA) occurs in approximately 50% of diagnosed amyloidosis cases [3] and generally develops in an aggressive form of heart disease with congestive heart failure and impaired ventricular filling, but usually with preserved ejection fraction [4]. Almost one third of AL amyloidosis cases are related to multiple myeloma. One of the most frequent complications in oncologic patients with AL amyloidosis is the development of atrial fibrillation (AF). These patients have a 4.4 times greater risk in developing AF than non-oncologic subjects in the first year since diagnosis [5].

In terms of prognosis, the median survival of AL amyloidosis is roughly six months from the onset of heart failure, if untreated [6,7]. Hence, the underlying importance of an early diagnosis, given that this category of patients has the highest morbidity and mortality among those diagnosed with amyloidosis [8].

## 2. Case Report

We report a case of a 56-year-old woman, known with hypertension and hypothyroidism, who was admitted for fatigue, exertional dyspnea, generalized edema, profuse sweating, and generalized decreased muscle strength, with the onset of signs and symptoms a few days prior to the admission. 

Ten months prior to admission, the patient had presented to an outside hospital with similar complaints. At that time, she was diagnosed with chronic heart failure but no etiology could be ascertained. Echocardiography revealed thickening of the left ventricular walls and a restrictive filling pattern. During the hospital stay, 900 mL of pleural fluid was drained from bilateral pleural effusions and intravenous diuretic treatment was initiated, followed by symptom remission. According to the family history, her father died suddenly at home. Her chronic treatment included beta-blocker, angiotensin-converting enzyme inhibitor, a loop diuretic, and levothyroxine. 

On the current presentation, the physical examination revealed a heart rate of 62 beats per minute, blood pressure of 150/106 mmHg, oxygen saturation of 97% in ambient air, respiratory rate of 26/min and jugular venous pressure of 8 cm H_2_O. No bruits were heard in the carotid, subclavian, or femoral arteries, and the peripheral pulses were 3+ throughout. The first and second heart sounds had regular rate and rhythm, and no gallops were audible. There was a grade 2/6 holosystolic murmur at the apex radiating towards the axilla and bilateral lower limb edema. The remainder of the examination was normal.

**Figure 1:**
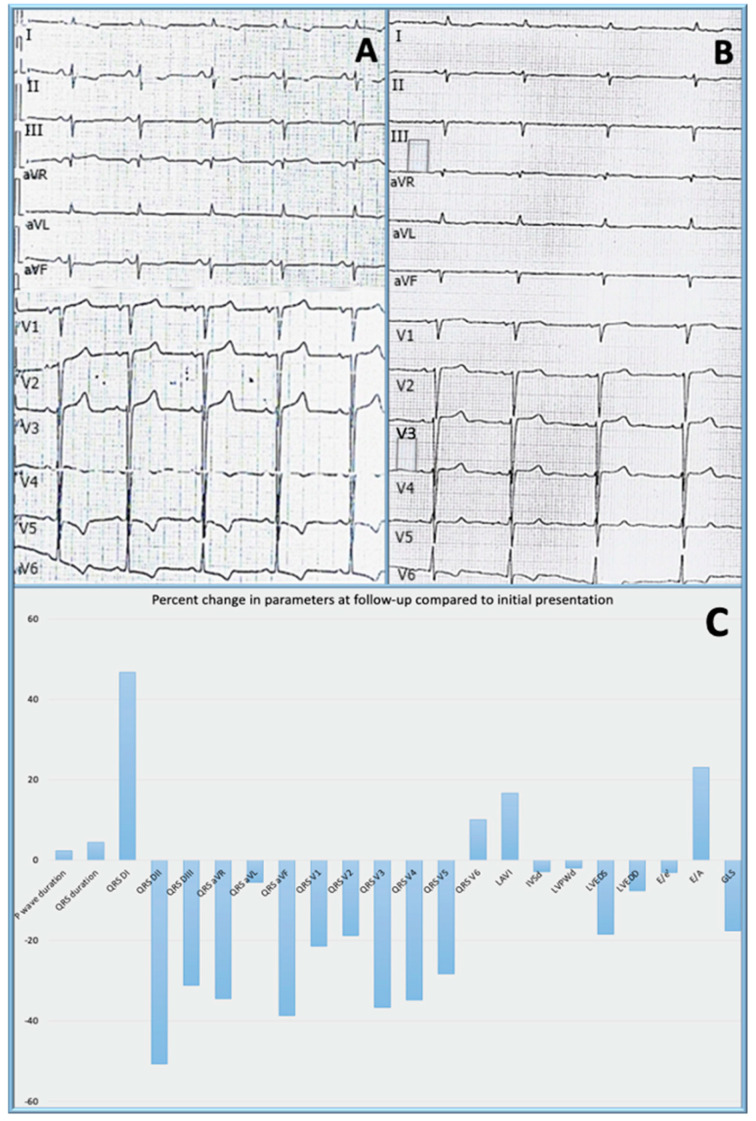
Electrocardiogram at baseline (**A**) and at follow-up (**B**) and percent changes in electrocardiographic and echocardiographic parameters at 9 months follow-up compared to the initial presentation; QRS DI − QRS V6 = QRS voltage in all 12 leads of the standard electrocardiogram; LAVI = left atrial volume index; IVSd = interventricular septum in diastole; LVPWd = left ventricular posterior wall in diastole; LVEDS = left ventricular end-diastolic diameter in systole; LVEDD = left ventricular end-diastolic diameter in diastole; E/e′ = mitral E/e′ ratio; E/A = mitral ratio between the maximal velocity of transmitral flow in early to late diastole; GLS = global longitudinal strain, presented as change in absolute value (**C**); QRS voltage measured as average of three consecutive beats, using digital calipers at 300% magnification calibrated for paper speed of 25 mm/s.

The electrocardiogram (Figure 1A) revealed sinus rhythm, with a ventricular rate of 62 beats per minute, QRS axis at −30°, left atrial enlargement, left ventricular hypertrophy (LVH) according to the Cornell criteria, poor R wave progression and negative T waves in lead I, lead II, aVL, V4–V6.

Laboratory tests (Table 1) revealed cholestasis with slightly increased liver enzyme levels, normal creatinine, slightly elevated urea nitrogen, leukocytosis, hyperkalemia, and N-terminal pro b-type natriuretic peptide (NTproBNP). A quantitative test for proteinuria was made, which revealed 241 mg proteins per 24 h. No other modifications were found.

**Figure 2:**
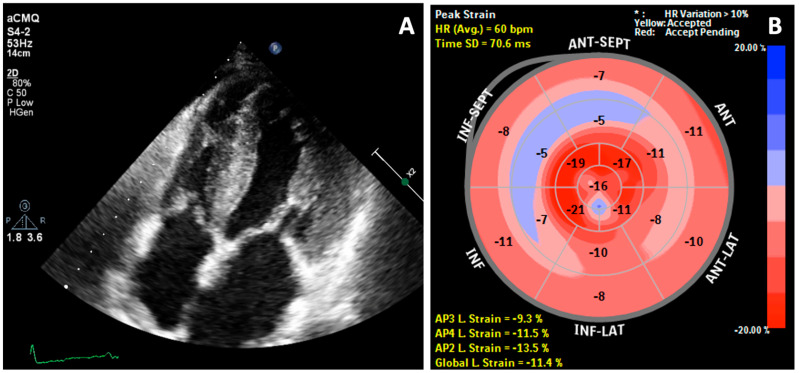
2D Transthoracic echocardiography: (**A**) Thickening of both atria, right ventricle walls, atrial septum, valves, and papillary muscles and (**B**) Reduced global longitudinal strain (GLS = −11.4%) with bull’s eye plot and pathognomonic pattern of relative apical sparing.

Transthoracic echocardiography (TTE) revealed a speckled hyperechoic appearance of the myocardium and symmetric LVH. In addition, there was thickening of atrial walls, right ventricular walls, atrial septum, valves and papillary muscles (Figure 2A). There was also biatrial enlargement and the left ventricular ejection fraction was preserved (52%). The diastolic function parameters were suggestive of elevated filling pressure. The mitral E/A ratio was 1.3. Tissue Doppler e′ velocity was reduced, with septal E/e′ ratio of 32.1 and lateral E/e′ of 17.8. Global longitudinal strain (GLS) imaging was −11.4%, with the bull’s eye plot (Figure 2B) revealing a ‘relative apical sparing’ characteristic for CA.

**Figure 3:**
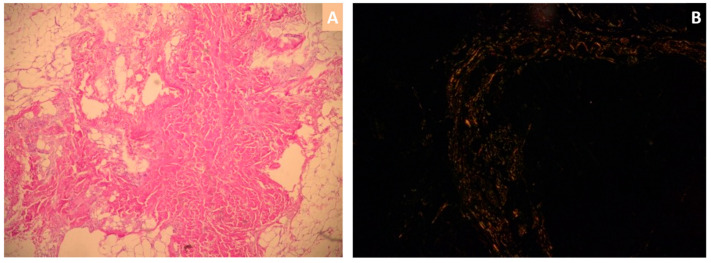
Abdominal fat pad biopsy: hematoxylin and eosin-stained biopsy sections revealing eosinophilic amorphous amyloid fibrils (**A**) and extracellular amyloid deposits with typical apple-green birefringence with Congo red dye under polarized light microscopy (**B**).

Native and contrast cardiac magnetic resonance imaging (cMRI) showed symmetric left ventricular wall thickening and preserved ejection fraction, which were consistent with the echocardiographic findings. The pattern of both diffuse subendocardial and transmural late gadolinium enhancement was seen in all four chambers of the heart, being suggestive for CA. Given this clinical presentation, it is not surprising that the abdominal fat pad biopsy revealed, on hematoxylin and eosin-stained biopsy sections, pink amorphous amyloid fibrils (Figure 3A) and apple-green birefringence with Congo red dye under polarized light microscopy (Figure 3B), thus confirming the diagnosis of systemic amyloidosis.

The immunofixation electrophoresis was positive for IgA and lambda light chain, urine protein immunofixation was positive for lambda light chain and the serum free light chain ratio was 0.05 (based on the elevation of lambda light chain). In the same time, bone tracer cardiac scintigraphy excluded transthyretin (ATTR) subtype given that no myocardial uptake of radiotracer was seen. 

Following the diagnosis path to establish the etiology of CA, bone marrow aspirate and biopsy were performed and revealed >25% large binucleated plasma cells. Immunoglobulin A type multiple myeloma with lambda light chain was diagnosed and the patient received specific therapy with bortezomib. At nine months after initiation of this therapy, the patient was seen in follow-up. She had no further hospitalizations for heart failure. However, the ECG voltage was found to be decreased (Figure 1B) when compared to the initial examination (Figure 1A) and the GLS on strain echocardiography was decreased as well. The percent changes in the ECG and echocardiographic parameters are presented in Figure 1C. Another ECG change at follow-up was the onset of AF, which was paroxysmal and was diagnosed on the 12-lead ECG. 

## 3. Discussion

Cardiac amyloidosis describes a rather underdiagnosed than rare condition, contrary to what it was presumed up until recently. The diagnosis of CA should be considered in any patient presenting with symptoms of congestive heart failure and evidence of restrictive cardiomyopathy [9].

We describe a case of a middle-aged woman with AL amyloidosis who had initially presented with dyspnea on exertion and lower extremity edema, ECG criteria for LVH, increased thickness of the LV walls and a restrictive pattern on echocardiography. In her case, natural disease progression was associated with significant reduction in QRS voltage and onset of AF. 

### 3.1. QRS Voltage

The QRS voltage in our patient has diminished significantly from the baseline examination. This phenomenon was identified in all ECG leads with the exception of lead I and V6 (Figure 1C).

**Figure 4:**
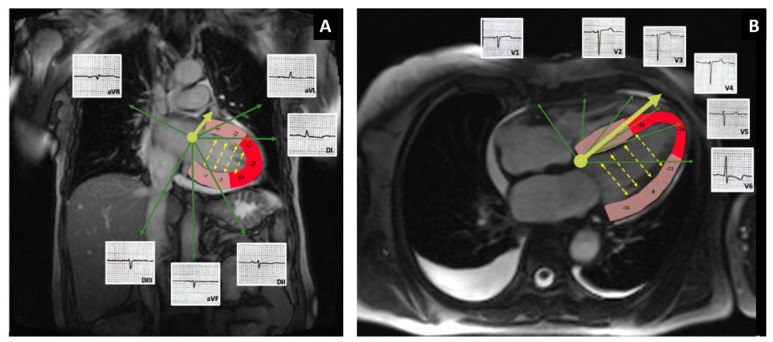
Potential mechanism responsible for the discordance in voltage between frontal (**A**) and precordial leads (**B**)**.** Fused cMRI-GLS images with examples of ECG waves on leads V1–V6. Segments of the LV are color-coded based on local strain values: red corresponds to local strain ≤−16%; pink local strain >−16%. The solid yellow arrow represents the QRS axis. The dotted yellow arrows represent direction of cancellation of electrical forces between opposing walls. The relative apical sparing pattern could be related to the high voltage seen in the precordial leads.

The most common ECG findings in CA patients are represented by low voltage with a pseudo-infarction pattern [10,11]. In contrast, our patient’s initial ECG revealed LVH, by Cornell criteria, with a poor R wave progression in the precordial leads. Although voltage criteria for LV hypertrophy are uncommon in patients with AL-CA, they can be present in up to 25% of patients with ATTR-CA. However, low QRS voltage was seen more often in subjects with AL-CA then in ATTR-CA [12]. There are cases described in the literature in which “true” LVH was generated by an overlapping hypertrophic cardiomyopathy [13]. In our patient, the voltage diminished in time so that criteria for low voltage was met in the frontal leads in spite of maintaining sufficient voltage in the precordial leads to fit the Cornell criteria for LVH. We do not have a clear explanation for this discordance between the voltage in frontal leads and precordial leads. A potential mechanism could involve the apical sparring encountered in CA in conjunction with the phenomenon of voltage cancellation. As seen in Figure 4, the LV apex and the periapical segments that have preserved strain values, being the proximity of leads V3 and V4, could still account for generation of enough voltage to meet the Cornell criteria for LVH (Figure 1B). 

The voltage along the short axis of the heart is diminished by the phenomenon of voltage cancellation (yellow dotted arrows), while the voltage along the QRS axis in the horizontal plane (yellow solid arrow, Figure 4B), and long axis of the ventricle, such as the voltage in lead V3 used for calculation of Cornell criteria for LVH, is less affected by this phenomenon of cancellation (no opposing walls along this axis). In frontal plane, the phenomenon is more prominent and therefore, the QRS amplitude meets criteria for low voltage. Conduction delay could be another mechanism that could potentially play a role in the generation of the QRS voltage and the LVH pattern [14]. In our patient, however, there was left anterior fascicular block at follow-up but the overall QRS duration remained unchanged compared to the baseline ECG.

The ECG findings are not diagnostic of CA, however, the association of low voltage on ECG with normal or high voltage in precordial leads and an increased thickness of LV walls on echocardiogram can raise the suspicion of amyloidosis [15]. Other echocardiographic findings that suggest CA, and were present in our patient are bi-atrial dilatation, thickening of the atrial septum, and thickening of the valvular leaflets. While CA is associated with diastolic dysfunction from early stages, LV systolic function remains within normal limits and starts deteriorating only at a later stage.

Another imaging modality used in the workup of patients suspected of CA is cMRI, which has significant value not only for the diagnosis of CA, but also for prognostic purposes, especially by defining the amyloid burden. In our patient, cMRI revealed both subendocardial and transmural late gadolinium enhancement (LGE). Fontana et al. [16] demonstrated that transmural or LGE observed at any level of the myocardium, is associated with the highest mortality ratio (hazard ratio, 4.1) and a median survival which usually does not exceed 17 months. Moreover, global pattern of LGE is also shown to provide incremental prognostics over biomarker stage [17]. Finally, cMRI is useful in recognizing concentric and symmetric LV thickening which tends to be far more frequent in AL amyloidosis then in ATTR amyloidosis (68% vs. 18%) [18,19].

### 3.2. Atrial Fibrillation

Given the association of CA with diastolic dysfunction, which ultimately leads to high ventricular filling pressures, AF is among the most frequent arrhythmias known to develop in patients with CA [11]. The prevalence was found to be 9% in patients with AL cardiac amyloidosis [20]. The risk of AF in patients with CA has been shown to be tightly related to three main predictors: GLS, left atrial volume index (LAVi), and the presence of left ventricular diastolic dysfunction. Impaired GLS (defined as GLS of greater than −14.7%) and abnormal LAVi (defined as a value greater than 31.0 mL/m^2^) are strong independent incremental predictors of AF with an HR/unit decrease of 1.20 and an HR/unit increase of 1.11 respectively. Besides, subjects with coexistence of both abnormal GLS and abnormal LAVi showed an incidence of new-onset AF >10-fold greater than those with both normal parameters [21]. Accordingly, our patient had a hazard ratio of 17.76 in developing AF with altered GLS, high LAVi and diastolic dysfunction. Given the presence of these risk factors, it is not surprising that at six months follow-up, she developed AF as natural progression of the disease. Regarding prevention of thromboembolic events, direct oral anticoagulants appears to be more effective than vitamin K antagonists (VKA), and one of the possible explanations is that a significant number of patients receiving VKAs experience difficulty in maintaining a therapeutic international normalized ratio (INR). In a recent study, Maurea et al. found that time in therapeutic range for INR is achieved in only a small percentage (12%) of the patients receiving treatment with a vitamin K antagonist [22].

### 3.3. Other Factors Related to Disease Progression in CA 

Other powerful prognostic factors found in our patient were the elevation in the NT-proBNP, and high concentration of circulating free light chain (FLC) proteins. Current Mayo stratification for CA segregates patients into four stages, based on serum levels of cardiac troponin T, NT-proBNP, and difference between involved and uninvolved FLC (dFLC). Based on this stratification, our patient was graded as stage IV with a 5-year overall survival of 14% and a median survival of 5.8 months [23]. Despite this, it is worth mentioning that our patient is still in observation 14 months since diagnosis. After specific treatment initiation, clinical status improved and dFLC was 0.01 mg/L, but cardiac function assessed by GLS at 9 months follow-up revealed a value of −9.7%.

Another hallmark of AL-amyloidosis is represented by the monoclonal peak of λ-FLC proteins from urine sample immunofixation which appears in the vast majority of cases. In our case, this monoclonal peak was absent, but Patel et al. showed that electrophoresis results are negative in about half of the times, even though monoclonal immunoglobulin or FLC are identifiable in the serum/urine in approximately 95% of patients with AL amyloidosis [24].

Echocardiography in CA often reveals the classical appearance of thickened ventricle walls with increased echogenicity and granular sparkling. Our patient presented the usual echocardiographic findings which were also consistent with the cMRI findings, but it is essential to notice that these are not specific to CA.

## 4. Conclusions

This case report presents a discordant electrocardiographic pattern of low voltage in the frontal leads and preserved voltage in precordial leads, in the context of a variety of clinical, laboratory, and echocardiographic abnormalities encountered during natural disease progression in a patient with AL amyloidosis.

The discordance between the voltage in the frontal leads and precordial leads, when present in conjunction with other findings, may be helpful in raising the clinical suspicion of cardiac amyloidosis.

## Figures and Tables

**Table 1:** Laboratory findings.

	On Presentation	Reference Range
Alanine aminotransferase (U/L)	44	<35
Aspartate aminotransferase (U/L)	48	<35
Alkaline phosphatase (U/L)	136	30–120
Glutamyl transpeptidase (U/L)	53	<38
Hemoglobin (g/dl)	14.7	12–15.5
Hematocrit (%)	44.9	37–47
White blood cell count (/mm^3^)	11.58	4–10
Platelet count (/mm^3^)	393	150–400
Sodium (mmol/L)	141	136–146
Potassium (mmol/L)	5.52	3.5–5.1
Chloride (mmol/L)	100	101–109
Urea nitrogen (mg/d)	69	17–43
Serum creatinine (mg/dl)	0.93	0.51–0.95
Glucose (mg/dl)	93	74–106
NTproBNP (pg/mL)	14.036	<125
Involved/uninvolved free light chains (dFLC) (mg/L)	296	

## Data Availability

Not applicable.
